# Regulation of Indole Signalling during the Transition of *E*. *coli* from Exponential to Stationary Phase

**DOI:** 10.1371/journal.pone.0136691

**Published:** 2015-09-02

**Authors:** Hannah Gaimster, David Summers

**Affiliations:** Department of Genetics, University of Cambridge, Downing Street, Cambridge, CB2 3EH, United Kingdom; University Paris South, FRANCE

## Abstract

During the transition from exponential to stationary phase *E*. *coli* produces a substantial quantity of the small, aromatic signalling molecule indole. In LB medium the supernatant indole concentration reaches a maximum of 0.5–1 mM. At this concentration indole has been implicated in many processes inducing acid resistance and the modulation of virulence. It has recently been shown that cell-associated indole transiently reaches a very high concentration (approx. 60 mM) during stationary phase entry, presumably because indole is being produced more rapidly than it can leave the cell. It is proposed that this indole pulse inhibits growth and cell division, causing the culture to enter stationary phase before nutrients are completely exhausted, with benefits for survival in long-term stationary phase. This study asks how *E*. *coli* cells rapidly upregulate indole production during stationary phase entry and why the indole pulse has a duration of only 10–15 min. We find that at the start of the pulse tryptophanase synthesis is triggered by glucose depletion and that this is correlates with the up-regulation of indole synthesis. The magnitude and duration of the resulting indole pulse are dependent upon the availability of exogenous tryptophan. Indole production stops when all the available tryptophan is depleted and the cell-associated indole equilibrates with the supernatant.

## Introduction

Indole is a small aromatic molecule produced by over 85 species of bacteria and implicated in multiple signalling processes [1]. Studies of indole signalling in *E*. *coli* have focused primarily on the effect of low (0.5–1 mM), persistent levels of indole, similar to those that are detected in an *E*. *coli* LB culture supernatant in stationary phase. At these concentrations indole has a variety of effects including modulation of biofilm formation, virulence and stress responses [2–4].

Higher levels of indole have very different effects. In particular, 4–5 mM indole added exogenously to an *E*. *coli* culture has been shown reversibly to inhibit growth and cell division [5]. Inhibition of cell division results from indole acting as a proton ionophore [6], reducing the electrical potential difference across the cytoplasmic membrane and preventing proper function of the MinCD system that positions the FtsZ ring [6]. The biological relevance of this effect was not immediately obvious because it requires indole at a concentration approximately 10-fold higher than that normally detected in the supernatant of stationary phase *E*. *coli* culture.

Recent work has shown that *E*. *coli* cells sometimes experience much higher indole concentrations but that such exposure is cell-associated and transient [7]. Indole production by *E*. *coli* is not constant during growth of a culture but occurs mostly during the transition between exponential and stationary phase (5) when the supernatant indole concentration increases 5-fold in approx. 30 min [7]. During this period indole is produced faster than it can exit the cell and consequently there is a rapid, but short-lived, rise in cell-associated indole. At its peak the cell-associated concentration reaches 60 mM, a level that would otherwise require the addition of 4 mM indole to the culture supernatant. This phenomenon is described by Gaimster et al. as the “indole pulse”. The effect of the pulse is to cause wild-type *E*. *coli* to enter stationary phase at a lower culture density than indole non-producing mutants. This conserves resources in the culture medium and results in a higher viability for wild-type cells during an extended stationary phase.

Here we investigate the mechanism of regulation of the indole pulse during the transition from exponential to stationary phase. What initiates rapid indole synthesis as stationary phase approaches and what is responsible for its rapid shut-down 10–15 min later? Indole is produced by the enzyme tryptophanase [8] that converts tryptophan to indole, pyruvate and ammonia. Tryptophanase is encoded by *tnaA* that is part of the tryptophanase operon, along with *tnaB* that encodes a tryptophan specific permease [9]. Transcription of the tryptophanase operon is tightly controlled, with catabolite repression [10–13] and transcriptional attenuation [14–15] playing key roles. When tryptophan levels are low, tryptophanase expression is repressed by premature termination of transcription, whereas when tryptophan levels are high, tryptophanase expression is induced. The interplay of these two controls means that when cells are growing in rich medium such as LB, indole is only detectable in the culture supernatant as the cells approach stationary phase [5,13].

## Results

### Increased tryptophanase expression correlates with the indole pulse

**Fig 1:**
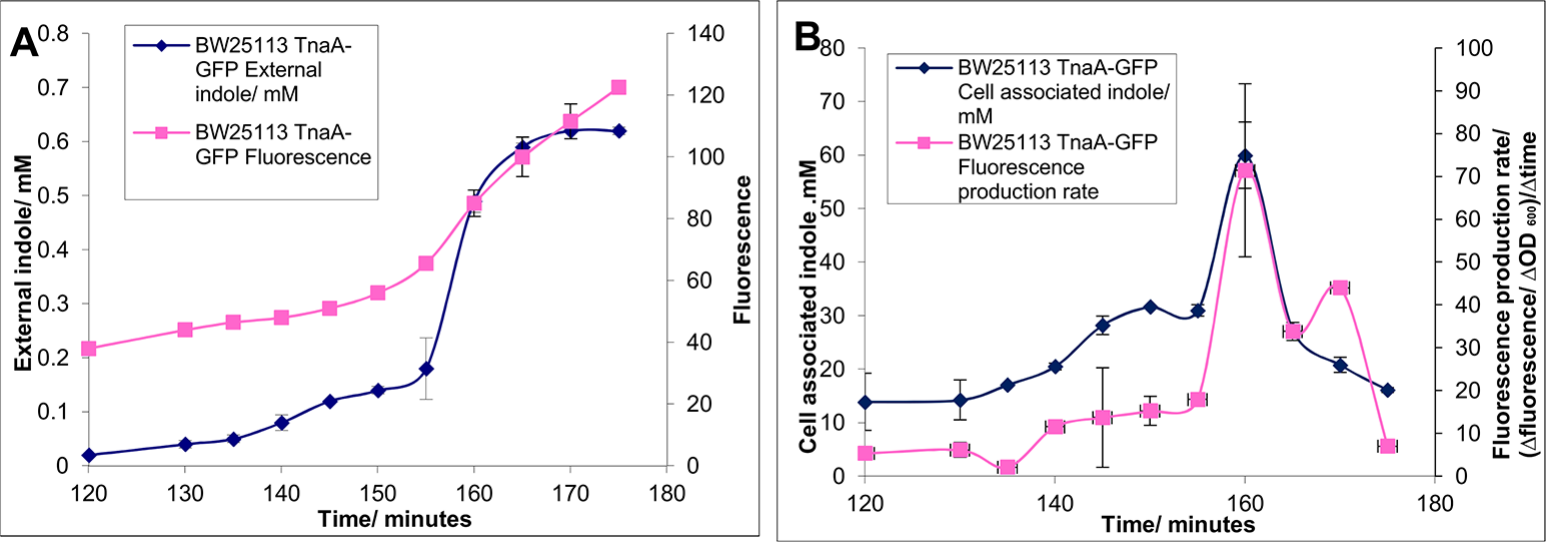
Tryptophanase expression increases rapidly at the same time as indole production. A culture of growing BW25113 TnaA-GFP cells in LB medium were sampled regularly. Panel A shows the increase in external indole as cell approach stationary phase (measured by Kovacs assay) and the raw fluorescence data (excitation 480 nm, emission 510 nm). Panel B shows the measured cell associated indole and calculated fluorescence production rate (Fluorescence production rate = (Δfluorescence/ ΔOD 600)/Δtime). Data shown are the mean values ± standard deviation for three independent repeats.

We investigated whether increased tryptophanase expression during the approach to stationary phase might be responsible for the initiation of the indole pulse. An overnight culture of BW25113 TnaA-GFP (complete list of strains used can be found in Table 1)was diluted into fresh LB medium to an OD _600_ of 0.05 (this was defined as t = 0; Fig 1) and grown at 37°C, with shaking. From t = 120 minutes, as the culture approached stationary phase, samples were removed at 5 minute intervals to measure external indole, cell-associated indole and tryptophanase expression (GFP fluorescence).

**Table 1:** List of *E*. *coli* strains used in this work.

BW25113	lacI^q^ rrnB_T14_ ΔlacZ_WJ16_ hsdR514 ΔaraBAD_AH33_ ΔrhaBAD_LD78_	Laboratory stock
BW25113 *tnaA*–GFP: Km^R^	lacI^q^ rrnB_T14_ ΔlacZ_WJ16_ hsdR514 ΔaraBAD_AH33_ ΔrhaBAD_LD78_ tnaA–GFP: Km^R^ introduced from MG1655 *tnaA*–GFP by P1 transduction Kanamycin resistant	This work
MG1655	F^−^ λ^−^ ilvG^−^ rfb-50 rph-1	Kindly supplied by Gang Li and Kevin D. Young Department of Microbiology and Immunology, University of Arkansas for Medical Sciences, Little Rock, AR 72205–7199, USA. Referenced in 13
MG1655 *tnaA*–GFP:: Km^R^	MG1655 *tnaA*–GFP Kanamycin resistance	As above

Tryptophanase-GFP fluorescence increased slowly until t = 155 min when the rate increased and doubled over the next 20 minutes (Fig 1A). However these fluorescence data are difficult to interpret because they represent tryptophanase per unit volume of culture under conditions when the number of cells per unit volume is increasing constantly. In order to see more clearly the changes in the production of tryptophanase per cell, we plotted the tryptophanase production rate (Fig 1B) ([change in fluorescence between successive samples/change in OD]/time between samples). In the early part of the experiment the production rate changed very little but at t = 155 there was a sudden 5-fold increase, followed by a decline.

In the same culture the supernatant indole concentration (Fig 1A) remained low (<0.2 mM) until t = 155 min when it began to increase rapidly, rising to a final concentration of 0.6 mM over the next 10 min. Cell associated indole (Fig 1B) peaked at approximately 60 mM at t = 160 min, before decreasing to approximately 10 mM, with the characteristic pulse kinetics described by [7]. There is a clear correlation between the time of increased TnaA expression and the accumulation of both cell-associated and supernatant indole. It seems reasonable to propose that this up-regulation of tryptophanase expression is primarily responsible for the increase in indole production during stationary phase entry.

### Glucose and tryptophan are key regulators of the indole pulse

Previous reports have demonstrated that tryptophanase expression is induced by tryptophan and subject to catabolite repression. We therefore investigated the effect of varying tryptophan and glucose concentrations on the expression of tryptophanase and the kinetics of indole production during stationary phase entry. To allow precise control of glucose and tryptophan concentrations the experiments were performed in minimal medium.

**Fig 2:**
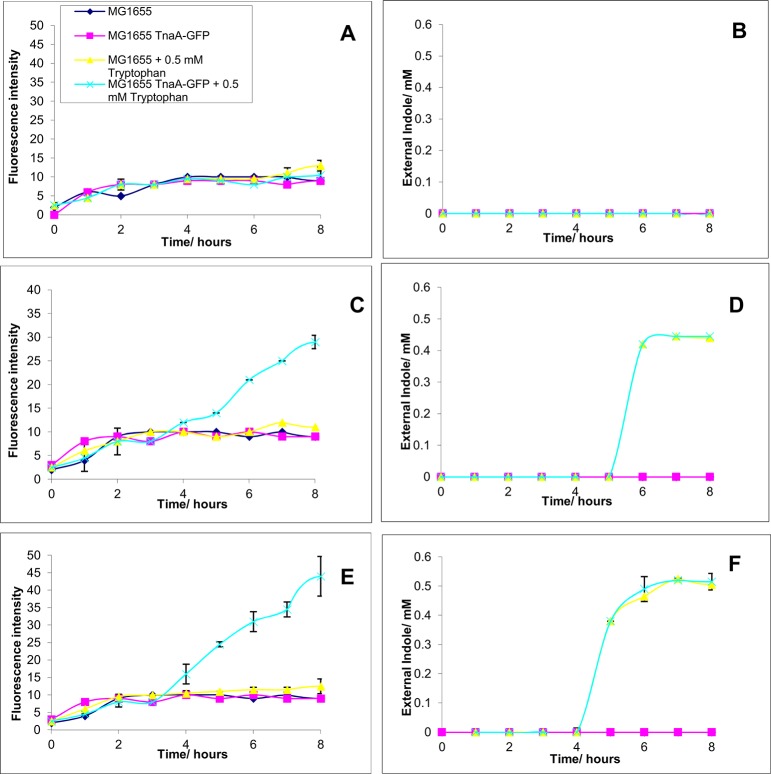
MG1655 and MG1655 TnaA-GFP grown in minimal medium with 0.2 (panels A+B), 0.1(C+D) or 0.05% (E+F) glucose produce TnaA and indole when 0.5 mM tryptophan is added. MG1655 and MG1655 TnaA- GFP cells were grown in minimal medium with 0.2, 0.1 or 0.05% glucose, with and without 0.5 mM tryptophan. Samples were removed hourly for 8 hours. The fluorescence intensity (excitation 480, emission 510 nm) (panels A, C, E) was measured and the supernatant was assayed for external indole using Kovacs assay (panel B,D F)). Data shown are the mean values ± standard deviation for three independent repeats.

Tryptophanase expression was measured using the in the Tna-GFP fusion strain MG1655 TnaA-GFP. Since the indole pulse was described initially in a different *E*. *coli* strain (BW25113) it was confirmed experimentally that neither growth (measured by OD_600_) nor indole production (measured by assaying supernatant indole) differed significantly between MG1655 and BW25113 cells (data not shown). Overnight cultures of MG1655 TnaA-GFP and MG1655 (TnaA^+^ but without a GFP tag) were diluted into fresh minimal medium (final OD_600_ = 0.5) supplemented with a varying concentration of glucose and with or without 0.5 mM tryptophan. These cultures were grown at 37°C with shaking and samples were removed every hour to assay GFP fluorescence and the supernatant indole concentration (Fig 2).

In medium containing 0.05% glucose, cells grew exponentially for approx. 4 h after sub-culture before entering stationary phase at OD_600_ = 1 (data not shown). The initial fluorescence intensity was low (<10 a.u.) and similar for all strains, irrespective of the presence of tryptophan or whether tryptophanase carried a GFP tag. After 3–4 h, fluorescence of the TnaA-GFP culture containing tryptophan began to increase above this background level, rising to approx. 45 a.u. over the next 5 hours (Fig 2E). There was no increase in fluorescence for the isogenic culture without tryptophan or for cultures where tryptophanase lacked the GFP tag. For the first 4 h after subculture indole was not detectable in the supernatant of any of the cultures. After 4 h, the supernatant indole concentration of the MG1655 TnaA-GFP and MG1655 TnaA^+^ (no GFP tag) cultures began to rise rapidly, reaching a maximum of 0.5 mM (Fig 2F). The two cultures showed indistinguishable indole production kinetics, confirming that the GFP tag on TnaA had no effect on the function of the enzyme. The period when the supernatant indole concentration is increasing rapidly (4–5 h after sub-culture) corresponds to the time of the indole pulse (Fig 1). As expected, in the absence of exogenous tryptophan no indole was produced.

The experiment was repeated with the glucose concentration increased to 0.1%. The cells grew exponentially for approx. 4 h after sub-culture before entering stationary phase at OD_600_ = 1. As observed for the culture containing 0.05% glucose, GFP fluorescence was seen only for the culture of MG1655 TnaA-GFP containing tryptophan (Fig 2C). Compared to the culture with 0.05% glucose, the time when GFP began to increase was slightly delayed, occuring 4–5 h after subculture. Indole production was also delayed and in the TnaA-GFP and MG1655 TnaA^+^ cultures supernatant indole began to increase rapidly after 5 h (Fig 2D).

Finally the experiment was repeated with the glucose concentration increased to 0.2%. This led to a substantial delay in stationary phase entry. The cultures grew exponentially until 4h after sub-culture, but did not reach their maximum density (OD_600_ = 2) until 8 h after sub-culture. There was no increase in GFP fluorescence in any culture for eight hours after sub-culture (Fig 2A). However, when the culture was checked after overnight incubation at 37°C, fluorescence of the MG1655 TnaA-GFP tryptophan-containing culture had risen to 25 a.u. (data not shown). Similarly, no supernatant indole was detected for 8 h after sub-culture (Fig 2B) but after overnight incubation the supernatant indole had reached 0.45 mM in the MG1655 TnaA-GFP and MG1655 TnaA^+^ cultures supplemented with tryptophan.

In summary, the experiments depicted in Fig 2 confirm that the period of rapid indole synthesis (corresponding to the cell-associated indole pulse) is coincident with the rise in tryptophanase activity and dependent upon the presence of tryptophan in the culture medium. When stationary phase entry is postponed by increasing the glucose concentration in the medium, the appearance of tryptophanase activity and the period of rapid indole synthesis are also delayed. These data are consistent with a simple model in which the rise in tryptophanase activity due to glucose depletion is the trigger for the indole pulse during stationary phase entry.

### Extra tryptophan amplifies the indole pulse

A change in tryptophanase activity can explain the start of the indole pulse but cannot account for its short duration. Although the enzyme is likely to remain active in the cell for an extended period, indole production ceases completely after approximately 30 minutes [7]. Li and Young (2014) have shown that the amount of indole produced by *E*. *coli* is determined by the amount of tryptophan in the growth medium [16]. Thus it is plausible that indole production slows and then stops as the supply of exogenous tryptophan is exhausted. Once the production rate falls below the rate at which indole diffuses from the cell, the cell-associated indole concentration will start to decline and the indole pulse will terminate.

**Fig 3:**
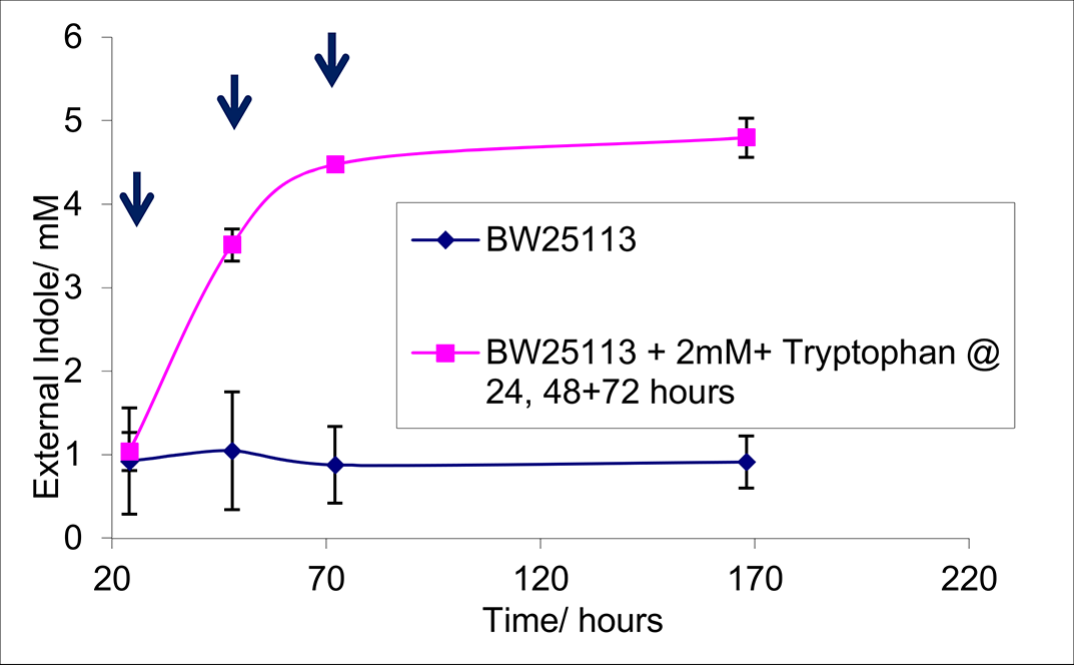
The addition of tryptophan to a stationary phase BW25113 culture leads to further indole synthesis. Stationary phase (24 hours) BW25113 cells were incubated with no additional tryptophan or 2 mM tryptophan added every subsequent 24 hours (arrows indicate times of addition). Samples were removed then centrifuged to remove cells. The supernatant was assayed for external indole using Kovacs assay. Data shown are the mean values ± standard deviation for three independent repeats.

In order to test the hypothesis that indole synthesis is limited by the availability of free tryptophan, an overnight culture of BW25113 cells was diluted into fresh LB medium to OD_600_ = 0.05. The culture was grown at 37°C with shaking for 24 hours. The supernatant indole concentration in the stationary phase culture was assayed and it was divided into two. One sub-culture was incubated at 37°C with shaking for a further 144 hours (6 days). The other culture was also incubated with shaking at 37°C but 2 mM supplements of tryptophan were added at 24, 48 and 72 hours. Thus, in total, a further 6 mM tryptophan was added to the culture. Samples were taken from the cultures every 24 hours for 72 hours and one final sample at 168 hours. The supernatant indole in each sample was assayed (Fig 3). After 24 hours, when no additional tryptophan had been added to either culture, the concentrations of indole in the culture supernatants were the same (approx. 1 mM). In the unsupplemented culture, the external indole remained constant at approximately 1 mM for 6 days. In the supplemented cultures, where an additional 2 mM tryptophan was added at 24 hours, the supernatant indole had risen to approximately 3.5 mM by 48 hours. This represents complete conversion to indole of the 2 mM tryptophan supplement in addition to approx. 1 mM present originally in the LB medium. A further 2 mM tryptophan was added at 48 hours and the supernatant indole had risen to approximately 5 mM by 72 hours. Once again, this represents almost complete conversion of the tryptophan supplement to indole. Although final supplement of 2 mM tryptophan was added at 72 hours, the external indole increased only slightly and remained at approximately 5 mM even after 168 hours. This is consistent with the findings of Li and Young (2014) who reported stoichiometric conversion of tryptophan into indole, up to a maximum indole concentration of approx. 5 mM. The data of Fig 3 demonstrate that active tryptophanase remains present in stationary phase cells for at least two days. During this period tryptophan availability limits indole synthesis. It therefore seems likely that tryptophan depletion is responsible for termination of the indole pulse during the transition to stationary phase.

**Fig 4:**
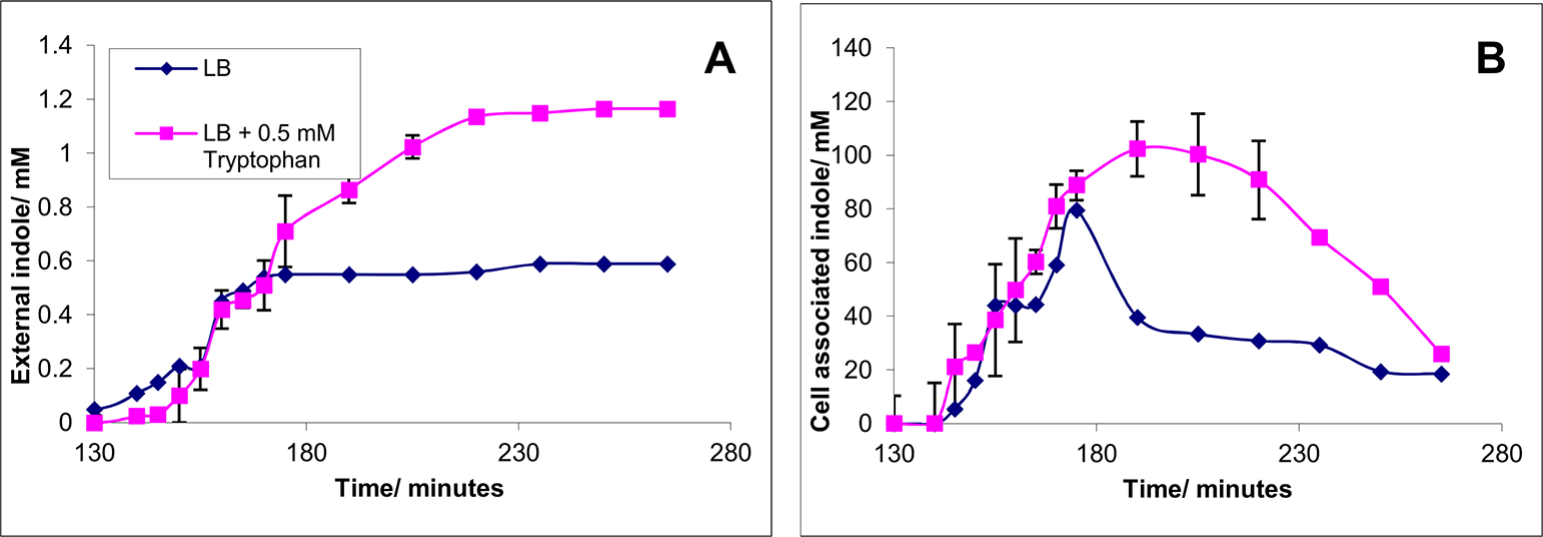
The effect of tryptophan supplementation on the indole pulse during stationary phase entry. A culture of growing BW25113 in LB medium with 0 or 0.5 mM tryptophan added was sampled regularly. The supernatant was assayed for external indole using Kovacs assay (panel A) and the cell pellet was assayed for indole using Kovacs assay (panel B). Data shown are the mean values ± standard deviation for three independent repeats.

To discover whether tryptophan availability determines the amplitude as well as the duration of the indole pulse we compared cells growing in LB with or without tryptophan supplementation. An overnight culture of BW25113 was diluted into fresh LB medium to an OD _600_ of 0.05. The culture was incubated at 37°C with shaking for 130 min at which point it was approaching stationary phase. The culture was divided into two, a tryptophan supplement (0.5 mM) was added to one sub-culture and both were incubated for a further 140 min. The concentration of indole in the supernatant and the cell associated indole in the samples were measured from the division of the culture (Fig 4).

When the culture was divided in late exponential phase (t = 130 min), the supernatant indole concentration was low (< 0.2 mM) but began to rise rapidly as stationary phase approached (Fig 4A). In both cultures the supernatant indole concentration rose to approx. 0.6 mM by t = 170 min. At this point indole production in the unsupplemented culture ceased, presumably because all available tryptophan had been converted into indole. In the culture with 0.5 mM tryptophan supplement, supernatant indole continued to increase for a further 50 minutes, eventually reaching approx. 1.1 mM. This reflects the conversion to indole of all the tryptophan present originally in the LB medium plus the 0.5 mM supplement.

Cell-associated indole was measured to discover the effect of the tryptophan supplement on the indole pulse (Fig 4B). In both the supplemented and unsupplemented cultures, cell-associated indole rose to approx. 70 mM by t = 170 min. For the unsupplemented culture this was the peak of the pulse and the cell-associated concentration then began to fall rapidly. In the supplemented culture cell-associated indole continued to increase for a further 45 minutes, reaching a peak of approx. 100 mM. Subsequently the concentration decreased but more slowly than for the unsupplemented culture. Thus the effect of tryptophan supplementation was to increase both the amplitude and duration of the indole pulse.

Tryptophanase expression is positively regulated by its substrate [14, 15]. We therefore investigated whether tryptophan supplementation in the previous experiment had increased tryptophanase activity and that this might be responsible for the altered kinetics of the indole pulse. An overnight culture of MG1665 TnaA-GFP was diluted into fresh LB medium (OD_600_ = 0.05) with or without a tryptophan supplement (0.5 mM). Samples were taken immediately and the fluorescence intensity measured. At this time the fluorescence intensity for the unsupplemented culture was 9±2 and the supplemented culture was 8±3.The cultures were then incubated at 37°C with shaking for 120 minutes. This took the cells to the transition between exponential and stationary phase when rapid indole production is observed. Further samples were taken to see if there was any difference in tryptophanase expression (GFP fluorescence) in response to increased tryptophan availability. At t = 120 min the fluorescence intensity for the unsupplemented culture was 26±2 and the supplemented culture was 27±4. Therefore a difference in tryptophanase expression cannot account for the change in the magnitude and duration of the indole pulse in the tryptophan supplemented culture.

## Discussion

Our results suggest a simple mechanism for regulation of the cell-associated indole pulse during *E*. *coli* stationary phase entry. We propose that in the presence of exogenous tryptophan, glucose depletion leads to increased tryptophanase expression, which in turn increases the indole production rate. Once the rate of indole production exceeds its rate of export, indole accumulates in the producer cells, resulting a rapid increase in cell-associated indole. When exogenous tryptophan is depleted indole production declines, even though active tryptophanase is still present in the cells. Once the rate of production falls below the rate of export, cell associated indole falls rapidly, eventually equilibrating with the concentration in the culture supernatant.

This model proposes that tryptophan depletion causes the end of the indole pulse. However, indole is a potent product inhibitor of tryptophanase [16] so could product inhibition of tryptophanase be alternative explanation? We see two main arguments against this. Firstly, when LB is supplemented with extra tryptophan, the cell-associated indole pulse is both higher and longer (Fig 4). Thus the cell must still contain active tryptophanase at the end of the normal pulse. Secondly, the maximum supernatant indole concentration obtainable by increasing exogenous tryptophan is approx. 5 mM (Fig 3) so tryptophanase must remain active up to this concentration. We have shown previously [7] that a supernatant concentration of 5 mM corresponds to a cell-associated concentration of approx. 80 mM, which is greater than the maximum cell-associated indole measured during stationary phase entry for cells growing in LB. However although the evidence argues against product inhibition being the main cause of the end of the pulse, it may still be a contributing factor.

To what extent is this model consistent with our existing understanding of indole signalling? Indole has been described in the literature as a quorum sensing molecule [1, 17]. This term was coined by Fuqua et al. (1994)[18], to describe a type of cell-to-cell communication, in which the expression of target genes is modulated in a cell density dependent way. An essential part of the quorum sensing paradigm is that conditioned medium added to low density cultures induces an effect normally seen at high density and this conditioned medium effect has indeed been reported for some examples of indole-inducible gene expression (4). In a similar vein, Lee and Lee (1) suggested that indole fulfils the 4 key criteria proposed by Winzer et al. [19] to be a ‘cell to cell signal molecule’ (CCSM). These are: (i) the CCSM is made at a specific stage of growth (ii) it accumulates externally and is recognised by a specific receptor (iii) the accumulation of the CCSM generates a concerted response and (iv) the response extends beyond physiological changes to remove or detoxify the molecule.

Indole pulse signalling during stationary phase entry meets some of these criteria but fails both the conditioned medium test for quorum sensing and the requirement of Winzer et al.[19] that the response is generated by an externally accumulated CCSM. Conditioning growth medium by adding 1 mM indole does not cause a tryptophanase-deficient strain to enter stationary phase with wild-type kinetics, nor does it restore viability in long-term stationary phase [7]. This is because cells are not responding to supernatant indole during stationary phase entry but to internal, or cell-associated indole.

Thus indole pulse signalling is not a community action but a cellular response to individual circumstances and this means that it is not limited to high density culture. In theory any one cell could potentially trigger a pulse of indole, irrespective of culture density, the behaviour of its neighbours or the supernatant indole concentration. This potentially provides a mechanism for indole production and cell division inhibition in cells undergoing a plasmid dimer catastrophe. Dimers of plasmid ColE1 accumulate rapidly by over-replication [20, 21]. and express a regulatory transcript, Rcd, that stimulates indole production by binding to the tryptophanase enzyme [5]. If indole is produced faster than it can escape from the cell, there will be an indole pulse that would reach a concentration sufficient to inhibit division of the producer cell. Since the end of the pulse occurs only when exogenous indole is exhausted, a small number of cells undergoing an Rcd generated pulse in a low density culture could maintain the pulse for an extended period. In the mean time the majority of cells in the culture would continue to grow and divide, and the concentration of indole in the culture supernatant would remain very low.

In conclusion this work reinforces our observations that there are two distinct modes of indole signalling, pulse signalling which demands a high, transient level of indole and persistent signalling which is due to low, long-lasting levels of indole. It is interesting to speculate that the confusion and contradictions within the indole signalling literature may, in part, be due to the fact that the effects of high, transient levels of indole have not been previously appreciated.

## Materials and Methods

### Microbiological techniques

Appropriate medium (LB: supplied by ForMedium or minimal medium (10 ml), with appropriate antibiotic if required, was inoculated with a single *E*. *coli* colony and incubated overnight with shaking at 120 rpm at 37°C. LB, lysogeny broth, is a nutritionally rich but chemically undefined medium. Overnight cultures were diluted appropriately and incubated with shaking at 120 rpm at 37°C. Growth was measured by monitoring optical density at 600 nm (OD_600_) using a Gene Quant 1300, GE Spectrophotometer.

### P1 Transduction

P1 transduction was used to transfer TnaA:GFP Km^R^ from MG1655 TnaA:GFP Km^R^ into BW25113 to create BW25113 TnaA–GFP: Km^R^

The experimental procedure consists of two parts.

#### 1. Preparation of donor lysate

A sample of overnight culture (500 ml) of the donor strain (i.e. the strain containing the marker to be moved) was added to 100 μl of phage suspension. CaCl_2_ was added to a final concentration of 10 mM. The mixture was left at room temperature for 5 minutes, before adding to 7 ml of soft agar (LB medium + 0.7% agar), prewarmed to 42°C. This mixture was then poured over an LA (LB medium + 1.5% agar) plate and incubated, with lids uppermost, overnight at 37°C. The following day the phage were harvested by scraping off the soft agar into a sterile tube. This was centrifuged at 2880 x g for 10 minutes at room temperature (Eppendorf 5810R Centrifuge). The mixture was transferred to a sterile Bijou tube, 500 μl of chloroform was added and the resulting phage suspension stored at 4°C.

#### 2. Transduction into recipient cells

An overnight culture of the recipient cells (i.e. the cells into which the marker is being moved) was diluted and allowed to grow to mid log phase (OD_600_ ~ 0.2). The cells were centrifuged at 2880 x g for 10 minutes at room temperature (Eppendorf 5810R Centrifuge). The resultant pellet was resuspended in 1 ml of absorption fluid (10:1 ratio of distilled water and LB + 1 mM CaCl_2_). The mixture was added to 150 μl of phage suspension and incubated at 37°C for 30 minutes. 10 μl of 5% (w/v) sodium citrate was added and the resultant mixture was spread onto selective plates containing 0.05% w/v sodium citrate. The plates were incubated overnight at 37°C. Colonies were purified of phage by streaking onto selective LA plates several times, and then screened for the required phenotype.

### Chemicals

All chemicals were purchased from Sigma. Minimal medium was made as follows 1x M9 salts, CaCl_2_ (0.1 mM) and MgSO_4_ (2 mM) in distilled water. Media was autoclaved before addition of appropriate concentration of glucose.

### Kovacs Assay

To assay indole in culture supernatants, a sample (1 ml) from a growing culture was removed, and cells harvested by centrifugation at 11337 x g for 15 seconds (Eppendorf Minispin microfuge). The supernatant was removed and assayed: 300 μl of Kovacs Reagent (10 g of *p*-dimethylamino-benzaldehyde dissolved in a mixture of 50 ml of HCl and 150 ml of amyl alcohol) was added to the supernatant and incubated for 2 minutes. A 50 μl portion was removed and added to 1 ml of HCl-amyl alcohol solution (75 ml of HCl and 225 ml of amyl alcohol). The absorbance at 540 nm was measured (Gene Quant 1300, GE Spectrophotometer). The concentration of indole in the supernatant was calculated using a calibration curve.

To assay cell associated indole, a modified method was used. A sample (1 ml) was taken from a culture, the OD_600_ measured and cells harvested by centrifugation at 11337 x g for 15 seconds. The supernatant was discarded and the cell pellet assayed: 300 μl of Kovacs Reagent was added to the cell pellet for 2 minutes. This lysed the cells and allowed the Kovacs reagent to react with indole. This mixture was pipetted into 1 ml LB before a 50 μl portion was removed and added to 1 ml of HCl-amyl alcohol solution. The absorbance at 540 nm was measured (Gene Quant 1300, GE Spectrophotometer). The concentration of indole was calculated using a calibration curve. This was converted to a cell associated indole concentration as described in [7].

### Fluorescence measurements

Samples were removed from growing cultures as required and the GFP fluorescence in the samples was measured by using 480 nm excitation and 510 nm emission (Cary Eclipse Fluorescence spectrophotometer). Using these data, the rate of change of fluorescence per cell for each strain was calculated. The change in fluorescence intensity between two consecutive samples was divided by the change in OD _600_ and the time elapsed.
